# Systematic analysis of the scientific quality, reliability, and educational value of Vaginal Natural Orifice Transluminal Endoscopic Surgery videos on YouTube

**DOI:** 10.1590/1806-9282.20260628

**Published:** 2026-10-12

**Authors:** Okay Alptekin, Betül Kalkan

**Affiliations:** 1 Erzincan Mengücek Gazi Training and Research Hospital, Department of Obstetrics and Gynecology – Erzincan, Turkey.; 2 Erzincan Binali Yıldırım University, Faculty of Medicine, Department of Obstetrics and Gynecology – Erzincan, Turkey.

**Keywords:** Natural orifice endoscopic surgery, Social media, Video recording, Education, medical, Gynecology, Reproducibility of results

## Abstract

**OBJECTIVE:**

The aim of this study was to evaluate the scientific quality, reliability, and educational value of YouTube videos on Vaginal Natural Orifice Transluminal Endoscopic Surgery using structured assessment tools.

**METHODS:**

This cross-sectional study analyzed 20 YouTube videos on Vaginal Natural Orifice Transluminal Endoscopic Surgery identified using the keywords "Vaginal Natural Orifice Transluminal Endoscopic Surgery," "Vaginal Natural Orifice Transluminal Endoscopic Surgery hysterectomy," "vaginal natural orifice endoscopic surgery," and "transvaginal NOTES." Two obstetrics and gynecology specialists independently evaluated the videos using the Global Quality Scale, modified DISCERN, and Journal of the American Medical Association benchmarks. Analyses included non-parametric group comparisons, Spearman correlation, multiple linear regression, and intraclass correlation coefficient analysis.

**RESULTS:**

The median number of views was 2,565 (interquartile range: 13,322). The mean Global Quality Scale, modified DISCERN, and Journal of the American Medical Association scores were 4.17±0.94, 3.95±0.84, and 2.65±0.93, respectively. Inter-rater agreement was moderate for Global Quality Scale and modified DISCERN and good for Journal of the American Medical Association. A statistically significant difference by channel type was observed only for modified DISCERN scores. Although Global Quality Scale scores were moderately correlated with likes (ρ=0.565, p=0.009), multiple linear regression showed no independent association between popularity or technical quality variables and quality scores (p>0.05).

**CONCLUSION:**

Vaginal Natural Orifice Transluminal Endoscopic Surgery videos on YouTube may provide valuable educational content; however, popularity metrics do not reflect scientific reliability. Video selection for surgical education should be based on structured quality assessment tools rather than view count alone.

## INTRODUCTION

Vaginal Natural Orifice Transluminal Endoscopic Surgery (vNOTES) provides vaginal access to the peritoneal cavity by combining endoscopic and laparoscopic methods, thereby avoiding an abdominal skin incision and potentially reducing perioperative morbidity^1^. It has been adopted as an alternative to conventional laparoscopy or open surgery in benign gynecologic procedures such as hysterectomy and adnexal surgery^2^. However, limited vaginal access, previous pelvic surgery, and advanced pelvic adhesions may restrict its applicability and require careful patient selection^3^.

Surgical education has evolved from the traditional master-apprentice model toward digital learning environments, and YouTube has become one of the most frequently used platforms for health-related and surgical educational content because of its open-access nature^4^. However, the absence of peer review raises concerns regarding accuracy: health-related and surgical videos are frequently incomplete, misleading, or heterogeneous in educational structure, presentation of technical steps, and source transparency^5,6^. Structured scoring systems have therefore been recommended to assess such material reliably^7^, and popularity metrics have not been found to reflect scientific quality across surgical fields^8,9^.

The literature has nevertheless focused mainly on common procedures, and studies evaluating YouTube content for relatively novel, technically demanding procedures with a pronounced learning curve, such as vNOTES, remain limited. vNOTES has a distinct learning curve due to its different anatomical orientation, limited instrument triangulation, and technical constraints in vaginal access^10^.

Studies evaluating the educational value of vNOTES videos remain limited, and the available evidence suggests that such content cannot be considered a reliable standalone resource for surgical education^11^. The present study differs from earlier YouTube analyses in three respects. First, it addresses a procedure still in its diffusion phase, for which structured proctorship is scarce, and trainees therefore rely more heavily on freely available video material. Second, educational quality, scientific reliability, and source transparency were assessed simultaneously with three complementary instruments by two independent and mutually blinded evaluators, with inter-rater agreement quantified rather than assumed. Third, the assumption that popularity indicates quality was tested in a multivariable model rather than by correlation alone. The aim of this study was to evaluate the scientific quality, reliability, and educational value of vNOTES videos on YouTube using structured assessment tools.

## METHODS

### Video screening strategy

This cross-sectional study was conducted in January 2026 and analyzed YouTube videos using the keywords "vNOTES surgery," "vNOTES hysterectomy," "vaginal natural orifice endoscopic surgery," and "transvaginal NOTES." Searches were performed in incognito mode to minimize bias. The first 50 results sorted by relevance for each keyword were screened. After duplicate removal, the remaining videos were assessed according to inclusion and exclusion criteria. Video selection was performed independently by two researchers, and disagreements were resolved by consensus.

### Inclusion and exclusion criteria

The study included videos directly related to vNOTES surgery that contained narration or subtitles in English or Turkish, allowed visualization of surgical steps, had a duration of at least 1 min, more than 100 views, and adequate image quality to clearly identify the surgical field (minimum 720p). Because vNOTES is a relatively recent surgical technique and the amount of available content is limited, a view-count threshold of more than 100 views was applied so that technically relevant videos would not be excluded.

Content intended for advertising or promotion, videos containing only patient experiences or interviews, theoretical presentations without surgical procedures, duplicate videos, and low-quality videos that were silent, lacked subtitles, or did not clearly show the surgical field were excluded. A total of 20 videos were included in the final analysis.

### Data collection and assessment scales

For each video, the following data were recorded: duration, upload date, views, likes, comments, language, channel type (academic journal, educational institution, or individual/medical channel), and whether source or reference information was provided in the description section. Data were collected using a standardized form. Video screening and scoring were performed by the two evaluators independently. Each evaluator was blinded to the other's scores, and no scores were shared or discussed until all evaluations were completed and the intraclass correlation coefficients had been calculated.

Both evaluators were blinded to video popularity metrics and scored the videos on content alone. The Global Quality Scale (GQS) assessed overall educational quality on a 1–5 scale, with higher scores indicating better quality.

The modified DISCERN (mDISCERN) scale assessed scientific reliability through five criteria: source citation, impartial presentation, consistency with current scientific evidence, discussion of risks and alternatives, and clarity; each was scored 1 for "yes," giving a total of 0 to 5.

The Journal of the American Medical Association (JAMA) criteria assessed transparency and academic accountability through authorship, source attribution, disclosure of conflicts of interest or sponsorship, and content currency, with one point per criterion and a total of 0 to 4.

### Statistical analysis

Statistical analyses were performed using IBM SPSS Statistics version 27 (IBM Corp., Armonk, NY, USA). Continuous variables were assessed using the Shapiro-Wilk test and were not normally distributed (p<0.05 for all variables). Therefore, data were presented as mean±standard deviation and median with interquartile range (IQR), and non-parametric tests were used for group comparisons.

The Kruskal-Wallis test was used to compare quality scores by channel type, and significant differences were examined with the Mann-Whitney U test. Relationships between continuous variables were assessed using Spearman's rank correlation coefficient (ρ).

Inter-rater agreement was assessed using the single-measure intraclass correlation coefficient (ICC [2,1]) based on a two-way random-effects model with absolute agreement and reported with 95%CI. ICC values were interpreted as <0.50, poor; 0.50–0.75, moderate; 0.75–0.90, good; and >0.90, excellent.

Multiple linear regression was performed with view count, like count, and video quality as independent variables and GQS, mDISCERN, and JAMA scores as separate dependent variables; multicollinearity, assessed by variance inflation factor, was within acceptable limits.

Two-sided p-values were used in all statistical analyses, and p<0.05 was considered statistically significant.

### Ethics statement

Ethics committee approval was obtained from the Erzincan Binali Yıldırım University Non-Interventional Clinical Research Ethics Committee (decision no: 2025-18/06; date: 16.10.2025). The study followed the Declaration of Helsinki and national ethical standards. As it analyzed only publicly available YouTube videos and involved no direct human participation or identifiable personal data, informed consent was not required.

## RESULTS

### Descriptive statistics and video characteristics

The main characteristics of the 20 included videos are presented in Table 1. The median video duration was 282.5 s (IQR: 290.8; range: 81–1920), the median view count was 2,565 (IQR: 13,322; range: 168–56,650), the median number of likes was 25.5 (IQR: 67.8), and the median number of comments was 0 (IQR: 1.0).

**Table 1:** General characteristics and descriptive statistics of the videos.

Variable	Mean±SD	Median (IQR)	Min–max
Duration (seconds)	433.2±470.5	282.5 (290.8)	81–1,920
Views	9,095±13,551	2,565 (13,322)	168–56,650
Likes	41.1±45.5	25.5 (67.8)	3–185
Comments	1.1±2.0	0.0 (1.0)	0–6
Mean GQS score	4.17±0.94	4.50 (1.5)	1.5–5.0
Mean mDISCERN score	3.95±0.84	4.00 (1.0)	1.5–5.0
Mean JAMA score	2.65±0.93	2.75 (1.5)	1.0–4.0

SD: standard deviation; IQR: interquartile range; GQS: Global Quality Scale; mDISCERN: modified DISCERN; JAMA: Journal of the American Medical Association.

Mean quality scores were 4.17±0.94 for the Global Quality Scale (GQS), 3.95±0.84 for mDISCERN, and 2.65±0.93 for the JAMA criteria (Figure 1).

**Figure 1:**
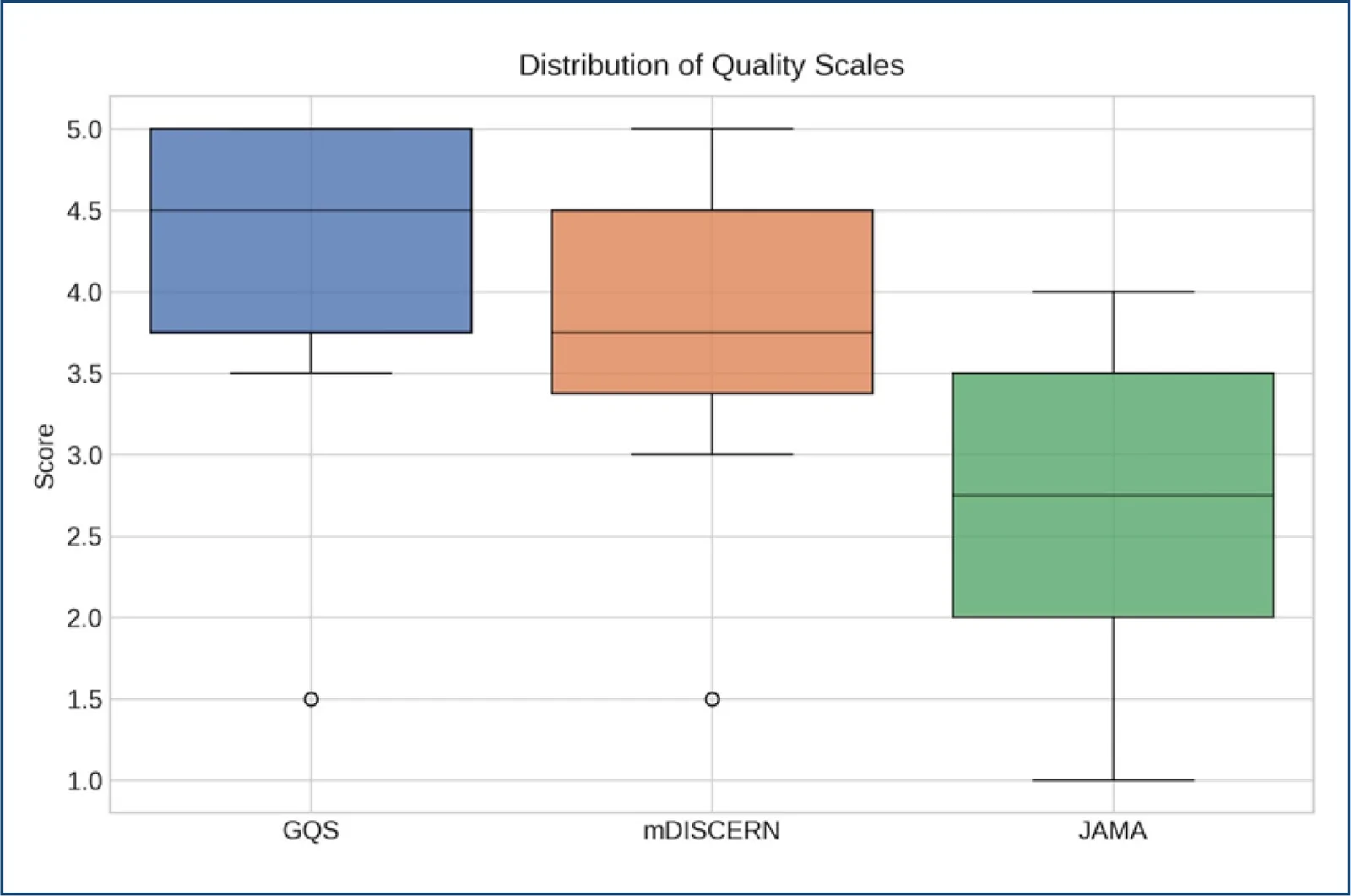
Distribution of Global Quality Scale, modified DISCERN, and Journal of the American Medical Association scores (box plots).

Seventy-five percent of the videos (n=15) were in English, and 25% (n=5) were in Turkish. According to channel type, educational channels constituted the largest group at 55% (n=11), followed by individual medical channels at 30% (n=6) and academic journal-based channels at 15% (n=3).

### Inter-rater reliability (intraclass correlation coefficient)

In the inter-rater reliability analysis between the two independent raters, good reliability was observed for the JAMA scale (ICC=0.706; 95%CI 0.40–0.87; p<0.001). Moderate reliability was observed for the GQS (ICC=0.609; 95%CI 0.25–0.82; p=0.001) and mDISCERN (ICC=0.518; 95%CI 0.12–0.78; p=0.007) scales (Table 2).

**Table 2:** Inter-rater reliability analysis (intraclass correlation coefficient).

Scale	ICC (single absolute)	95%CI	p-value	Level of agreement
GQS	0.609	0.25–0.82	0.001	Moderate
mDISCERN	0.518	0.12–0.78	0.007	Moderate
JAMA	0.706	0.40–0.87	<0.001	Good

ICC: intraclass correlation coefficient; CI: confidence interval; GQS: Global Quality Scale; mDISCERN: modified DISCERN; JAMA: Journal of the American Medical Association.

### Comparisons by channel type and language

A statistically significant difference by channel type was observed only for mDISCERN scores (Kruskal-Wallis, p=0.041). Journal-based channels had higher mDISCERN scores than educational and medical channels. However, this group comprised only three videos, and this finding should therefore be interpreted with caution. No significant differences were found for GQS (p=0.083) or JAMA scores (p=0.483). No statistically significant differences were found between Turkish and English videos for GQS, mDISCERN, or JAMA scores (p>0.05 for all comparisons).

### Correlation and regression analyses

In the Spearman correlation analysis, a moderate positive correlation was found between GQS scores and views (ρ=0.490, p=0.028) and likes (ρ=0.565, p=0.009). In contrast, no significant relationship was found between mDISCERN and JAMA scores and views, likes, comments, or video quality (p>0.05). In multiple linear regression analysis, none of the models, including view count, like count, and video quality, were significantly associated with GQS, mDISCERN, or JAMA scores. The GQS (R^2^=0.312, p=0.104), mDISCERN (R^2^=0.238, p=0.214), and JAMA models were all non-significant, and only overall model fit is therefore reported.

## DISCUSSION

The findings showed that vNOTES-related YouTube videos generally had good educational quality (GQS: 4.17±0.94) and satisfactory scientific reliability (mDISCERN: 3.95±0.84), but only moderate transparency (JAMA: 2.65±0.93). These findings differ from systematic reviews describing surgical YouTube videos as low quality. Gorgy et al. reported that 96.6% of surgical videos were educationally unsatisfactory^9^, and health-related YouTube content in general has been judged to remain below acceptable standards^4^. In the only previous vNOTES-specific analysis, Temur reported that 43.1% of videos were low quality and that the median GQS score was 2^11^.

This may be explained by the inclusion criteria applied in our study, which required a minimum resolution of 720p, a minimum duration of 1 min, and clear visualization of the surgical field. These criteria may have improved the overall quality of the sample by excluding low-resolution or amateur content that could contribute to misinformation. In addition, 15% of the videos were sourced from academic journals and 55% from structured educational channels, which may have contributed to the higher reliability observed in journal-based channels (p=0.041). However, only three videos originated from journal-based channels, and this observation should therefore be regarded as preliminary. Comparable observations have been reported for other gynecologic procedures, where videos uploaded predominantly by healthcare professionals nevertheless achieved only moderate overall quality and low mDISCERN scores because of insufficient academic referencing^12,13^. These findings suggest that high visual quality, reflected by GQS, does not always correspond to high scientific reliability, reflected by mDISCERN.

Videos produced by academic institutions, professional organizations, or surgeons have consistently been reported to provide higher educational value and transparency than those from individual users^6,9,11^.

One of the main findings of our study was that view and like counts had no independent effect on quality scores in multiple linear regression analysis. Assuming that the most viewed video is also the most reliable may therefore be misleading, as platform algorithms prioritize engagement over accuracy and allow scientifically flawed techniques to reach large audiences^4^. Therefore, structured assessment tools such as GQS, mDISCERN, and JAMA should be prioritized over popularity metrics in surgical educational materials.

vNOTES has a distinct learning curve because of its reverse anatomical perspective and limited instrument movement, with approximately 16 cases required for operative stability and 18 for proficiency in vNOTES hysterectomy^10^. However, Kapurubandara et al. emphasized that attempting innovative techniques such as vNOTES based solely on YouTube videos may involve serious surgical risks without structured proctorship training^3^. The educational value of video materials depends on clear demonstration of surgical safety, including the Critical View of Safety; however, compliance with these standards has generally been reported to be below 10%^9^.

The study was limited by the small sample of 20 videos, which restricts generalizability, and by the dynamic nature of YouTube content, meaning that the data reflect a single time point^11^. The sample was small because the number of vNOTES videos meeting the predefined technical and content criteria was limited; findings based on 20 videos, and in particular subgroup comparisons involving as few as three videos, should therefore be regarded as descriptive and hypothesis-generating rather than definitive. In addition, the inclusion criteria applied here (minimum 720p resolution, minimum 1-min duration, and clear visualization of surgical steps) are likely to have preferentially selected structured, institutional, or professionally produced content. The analyzed sample therefore represents a preselected, comparatively high-quality subset rather than the full spectrum of vNOTES content on YouTube. This may explain the higher scores observed compared with broader YouTube analyses and limits the generalizability of our findings. Peer-reviewed official video channels created by surgical societies and academic institutions may improve digital medical education and help counter anecdotal bias on YouTube^4^. For surgical education, these findings support a practical approach: video material should be selected by structured criteria rather than view count, preferably from accredited society or journal channels; it should complement rather than replace proctored hands-on training; and trainees should verify authorship, referencing, and disclosure before adopting a technique observed online. Curated, peer-reviewed vNOTES video libraries in which safety-critical steps are explicitly demonstrated would support this.

## CONCLUSION

Within this preselected sample, YouTube videos on vNOTES provided visually adequate educational content, although quality varied considerably. Videos from academic journal-based sources tended to show the highest reliability scores; however, this group comprised only three videos and the observation requires confirmation in larger samples. Because view and like counts are insufficient indicators of quality, structured assessment tools such as GQS and DISCERN should be used in selecting videos for surgical education. As our inclusion criteria yielded a technically preselected subset, these findings cannot be generalized to all vNOTES content on YouTube. Accredited channels endorsed by surgical societies may further improve the reliability of digital medical education.

## Data Availability

The datasets generated and/or analyzed during the current study are available from the corresponding author upon reasonable request.
